# Development and In Vitro Assessment of a Novel Vacuum-Based Tissue-Holding Device for Laparoscopic and Robotic Kidney Cancer Operations

**DOI:** 10.3390/cancers14194618

**Published:** 2022-09-23

**Authors:** Michael Gabi, Uwe Bieri, Venkat Ramakrishnan, Tilo Niemann, Antonio Nocito, Nadine Brader, Caroline Maake, Lukas John Hefermehl

**Affiliations:** 1 Independent Researcher, Kawasaki 210-8577, Japan; 2 Division of Urology, Department of Surgery, Kantonsspital Baden, 5404 Baden, Switzerland; 3 Division of Urology, Brigham and Women’s Hospital, Harvard Medical School, Boston, MA 02115, USA; 4 Institute for Radiology, Kantonsspital Baden, 5404 Baden, Switzerland; 5 Department of Surgery, Kantonsspital Baden, 5404 Baden, Switzerland; 6 Institute for Anatomy, University of Zurich, 8057 Zurich, Switzerland

**Keywords:** holding device, laparoscopy, robotic surgery, partial nephrectomy, vacuum

## Abstract

**Simple Summary:**

Depending on the location and configuration of renal tumors, it is sometimes difficult to grasp, excise, and properly remove them. We have designed and developed a device to improve robotic laparoscopic partial nephrectomy. We tested the device on porcine kidneys and livers and embalmed human cadavers. A preliminary evaluation of this surgical tool showed satisfactory device setup, suction, and tissue handling characteristics.

**Abstract:**

In this paper, we describe the development and evaluation of a novel tissue-holding device (THD) for use during robotic-assisted laparoscopic partial nephrectomy. The THD is a vacuum-based apparatus made of either 3D-printed polyethylene or stainless steel. The proximal end connects to suction tubing routed outside the body, while the distal end is conically shaped and designed to firmly interface with the tumor. Device feasibility studies were performed on six porcine kidneys, two porcine livers, and two embalmed human cadavers. A Likert-scale rating was used to assess device setup, suction, and tissue handling. Additional tests were performed using the daVinci Xi® robotic system. Finally, the holding force of the THD was assessed using different standard vacuum systems and pressure settings. In porcine tissue, the device setup, tissue suction, and handling were rated as “good”. THD insertion and removal was uncomplicated. In a simulated transabdominal approach on fixed human cadavers, the device setup, suction, and tissue handling were also rated as “good”. No macroscopic tissue compromise or device deterioration was noted. The handling and holding abilities using the daVinci Xi® robotic system were also rated “good”. The device was able to successfully hold over 300 g of tissue at a suction pressure of −600 mmHg. The preliminary evaluation of the THD demonstrated satisfactory results.

## 1. Introduction

Laparoscopy and robotic surgery have become the standard approach for many operations [1,2]. Patients undergoing laparoscopic partial nephrectomy (LPN) for localized kidney tumors experience decreased blood loss, reduced pain, and improved recovery when compared with open surgery [3,4,5]. However, grasping and manipulating the tissue parenchyma with laparoscopic and robotic instruments without causing tissue damage can be challenging. In the context of an operation to remove a tumor, improper tissue handling can result in significant bleeding [6], increased operative time and anesthesia exposure [7], and the potential seeding of tumor cells in the operative field [8]. Though there are a wide variety of laparoscopic and robotic instruments that aid in tissue grasping and dissection, to our knowledge, none exist for the expressed purpose of gently handling fragile or diseased parenchyma. We sought to develop an instrument that could be used trans- or retroperitoneally in a laparoscopic or robotic setting. Such a device would need to be easily inserted through conventional trocars and manipulated with standard laparoscopic graspers. The device would allow the surgeon to gently grasp bulky, smooth, and curved tissues, such as kidneys or liver, without significant tissue deterioration. Here, we describe the development and evaluation of a novel vacuum-based laparoscopic tissue-holding device (THD) with the aforementioned attributes. The study aimed to establish device feasibility and safety prior to further in vivo testing in animal and human trials.

## 2. Materials and Methods

### 2.1. Device Prototyping

THD prototypes were designed using 3D-printing in either polyethylene (prototype #1) or stainless steel (prototype #2) configurations (Proto Labs Germany GmbH, Feldkirchen, Germany). 

### 2.2. Tissue-Holding Experiments

Vacuum suction testing was performed using standard 4 mm urethane connecting tubes (UB0425.5/32; t313046, Pisco Pneumatic Equipment, Nagano, Japan) and with two standard OR vacuum devices—one electric pump (Medela AG, Baar, Switzerland) and a wall-based vacuum regulator (Greggersen Gasetechnik GmbH, Hamburg, Germany).

Different suction settings were assessed on porcine kidneys using a Profiscale high-performance digital spring balance (Tara PS 7620, Burg-Wächter, Wetter, Germany) and the OR vacuum suction devices. Porcine kidneys were placed in-line with an empty semi-flexible infusion container that could be filled with water and the spring balance. The device’s ability to lift the tissue was tested using an electric pump (with pressures spanning −200 mmHg to −600 mmHg) or a wall regulator (pressure of −600 mmHg). At each of these pressures, the maximal mass (grams, [g]) of the lifted tissue at the time of device-tissue disconnection was recorded. In the event the entire organ was lifted, the infusion container was filled with an additional quantity of water until disconnection occurred. In this instance, the additional filling volume (mL) was converted to a mass (g) and added to the tare weight of the organ. Macroscopic tissue impairment was reported as a binary outcome (yes/no) and was subjectively determined by the surgeon’s visual assessment.

### 2.3. Conventional and Robotic Laparoscopy Experiments

For laparoscopic and robotic tests, a standard wall-based vacuum suction regulator (Greggersen Gasetechnik GmbH) similar to those found in many operating rooms (ORs) was used with a standard setting of −600 mmHg. Conventional laparoscopic experiments were performed using a standard laparoscopy setup with laparoscopic graspers and scissors. Robotic-assisted laparoscopy experiments were carried out using a daVinci Xi® system (Intuitive Surgical Inc., Sunnyvale, CA, USA) at a local Intuitive test center in Switzerland. Grasping studies were performed using robotic Fenestrated Bipolar and ProGraspTM forceps (Intuitive Surgical Inc.). All laparoscopic and robotic experiments were performed by an experienced urologic surgeon (L.J.H.) and observed and evaluated by an experienced engineer (M.G.). Animal feasibility studies were performed using fresh, non-fixed porcine kidneys and livers purchased from a local slaughterhouse. Human feasibility studies were performed on Thiel-fixed human cadavers with study foci on the kidneys and liver. Human studies were carried out at the Anatomical Institute of the University of Zurich, and all human tissue was maintained and stored in accordance with the guidelines of the University. THDs were assessed laparoscopically as per their ability to (a) be held by a conventional laparoscopic grasper, (b) lift organs without dropping them, (c) handle tissue in horizontal and vertical planes, and (d) handle simulated tumors in the context of facilitating tumor dissection, extrication, and deposition into a laparoscopic Endo-catch bag. THDs were assessed on both porcine and human kidneys and livers. The device’s abilities were assessed and reported on a subjective 3-point Likert scale (1 = “bad”, 2 = “marginal”, and 3 = “good”). Each organ was assessed twice. (Supplementary File S1: Experiment documentation form).

## 3. Results

### 3.1. THD Development Process

**Figure 1:**
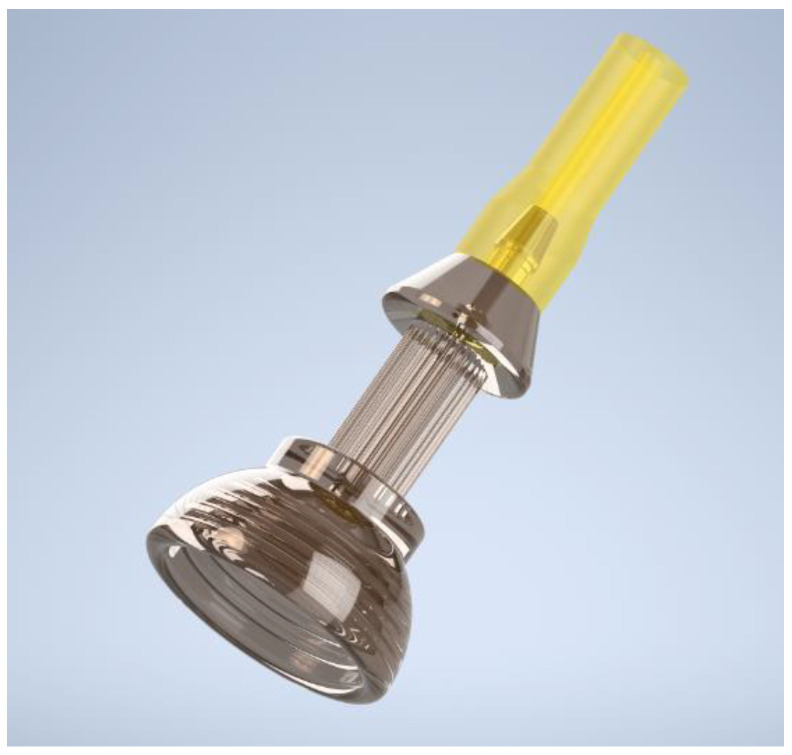
Prototype #2 of THD produced with stainless steel. The conical end is doom shaped for optimal contact with the tissue, the upper pole is designed to firmly connect with a hose. The inner bore at the level of the hose connection was widened to 1.8 mm, and the retention grid was removed.

The proximal end of the THD was designed to accommodate a small hose with an inner diameter of 2 mm and interface with standard OR suction equipment. The distal end of the THD is conical, with a maximal diameter of 10 mm and a rigid, dome-shaped retention grid designed to interface with and firmly hold onto tissues. The small diameter allows an insertion of the device via the 12 mm assistant-port at any time point. The first THD prototype was produced out of 3D-printed polyurethane. Its use in the conventional laparoscopic model setting (described below) ultimately resulted in the production of a second prototype made of stainless steel with modified dimensions (Figure 1). In the second iteration, the inner bore that interfaced with suction tubing was widened to 1.8 mm from 0.5 mm, and the inner diameter of the tube was changed from 2 mm to 3 mm. These modifications prevented the hose from clogging and facilitated the application of an increased vacuum force on the tissue. Moreover, the retention grid in the first prototype was removed in the second; its inclusion made it more difficult to precisely grasp the device by requiring the user to grasp a small protrusion (prototype #1) instead of the outer shell of the device (prototype #2). 

### 3.2. THD Prototype #1 Feasibility Tests

#### 3.2.1. Experiments in the Conventional Laparoscopic Model Setting

At a suction pressure of −600 mmHg, all of the kidneys (*n* = 3) were completely lifted off the testing surface and none were dropped, indicating that prototype #1 could form a tight seal with the smooth surface of an organ. All tissues were assessed macroscopically afterward, and no tissue deterioration was noted. 

Secondly, prototype #1 could be firmly grasped using laparoscopic graspers (*n* = 6) without loss of control of the device and was rated as “good”. 

Tissue handling with the attachment of the THD to the kidney tissue with a laparoscopic grasper in the horizontal and vertical plane (*n* = 6) was rated 3 on the Likert scale (“good”). The final experiment, labeled "tumor dissection," was designed to evaluate all of the features examined individually in the previous experiments in a combined sequence. 

Tumor dissection was simulated with the resection of three randomly selected circular-shaped tissue islets containing cortex and medulla structures on the anterior and posterior surface of three kidneys (*n* = 18). The tissue islets were consecutively transferred into a standard laparoscopic removal bag. Disconnection from the THD was realized by applying positive air pressure to the hose from the outside distal end via a standard 10 mL syringe. This standard operative sequence could be performed without interference. All sequential sub-steps of this task (parameters: holding abilities, handling of THD, tissue disconnection, and dropping to bag) were rated 3, i.e., “good” on the Likert scale, by both researchers (L.H. and M.G.).

The experiments were repeated on one liver to assess if the different parenchymatous structure of the liver tissue had a relevant influence on the tested properties of the device. The holding experiments (*n* = 2), grasping experiments (*n* = 2), handling experiments (*n* = 2), and tumor dissection experiments (*n* = 6) all led to the same Likert score rating of 3, i.e., “good”, as in the experiments with the kidneys. 

Despite the good results of the first series of tests, some minor flaws were identified (see evolutionary development process), which led to the development of prototype #2, which was tested in the subsequent experiments.

#### 3.2.2. Evaluation of Tissue Damage

We carefully performed macroscopic evaluations of the resected tissue as well as the surrounding parenchyma and we experienced no tissue damage.

### 3.3. The Experiments Described in the Following Were Performed with THD, Prototype #2

#### 3.3.1. Conventional Laparoscopy in Thiel-Fixated Human Bodies (Kidney and Liver)

Based on the experience from the experiments in the laparoscopic model settings, the setup was transferred to Thiel-fixated human bodies. According to the protocol, the first item evaluated was the introduction of the THD through a transperitoneal-placed 12 mm trocar. This task was performed (*n* = 6) without any problems and rated 3 (“good”) on the Likert scale. This was followed by evaluating the handling abilities and perceived dexterity of the THD with a laparoscopic grasper (*n* = 6) by moving the THD in the anterior-posterior and the medio-lateral axis inside the peritoneal cavity while simultaneously performing rotating motions from the wrist. During the initial sequence of the tissue connection/holding/manipulation test (*n* = 1), it was noticed that due to the nature of the Thiel-fixation, a significant amount of an oily-like fluid covering all inner organs led to an insufficient connection of the device. Therefore, the Likert scale rating of the initial experiment was 1 (“bad”).

The experiment was repeated after wiping and drying off the kidney surface with a paper towel. This led to significant improvement of the holding abilities of the THD, and the performance of the THD for the subsequent runs (*n* = 5) were all rated 3 (“good”). 

Finally, tumor dissection as described in the laparoscopic model setting was performed (*n* = 6) and was also rated 3 (“good”). Again, all experiments were also performed on the liver (*n* = 2) and led to the same ratings (3, “good”) for all experiments. 

#### 3.3.2. Robotic-Assisted Laparoscopy (Davinci Xi® System) Experiments with Porcine Kidneys (and Liver)

**Figure 2:**
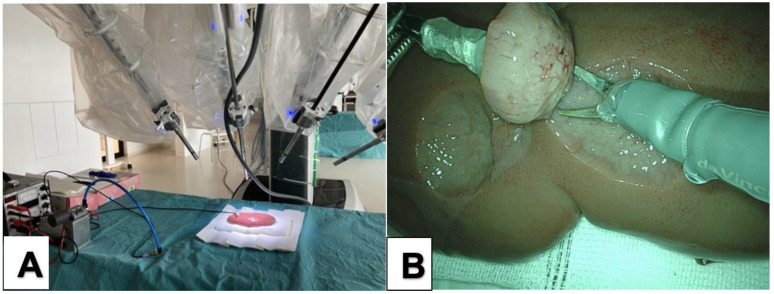
(**A**) Setup of the experiments with the daVinci Xi® system. (**B**) the THD was manipulated with the “Pro Grasp” Instrument, and resection was performed with the “Monopolar Scissors”.

To be sure that manipulation with the THD was also feasible in a robotic setting, we performed in vitro tests with porcine kidneys (*n* = 3) and liver (*n* = 1) using a daVinci Xi® system (“Pro Grasp”, “Maryland”, “Monopolar Scissors”) (Figure 2). Again, all parameters were rated as “good”. We experienced the same satisfying effect as seen in the conventional laparoscopic tests earlier. 

#### 3.3.3. Holding Force Measurements Using Different Vacuum Settings in Porcine Kidneys 

**Figure 3:**
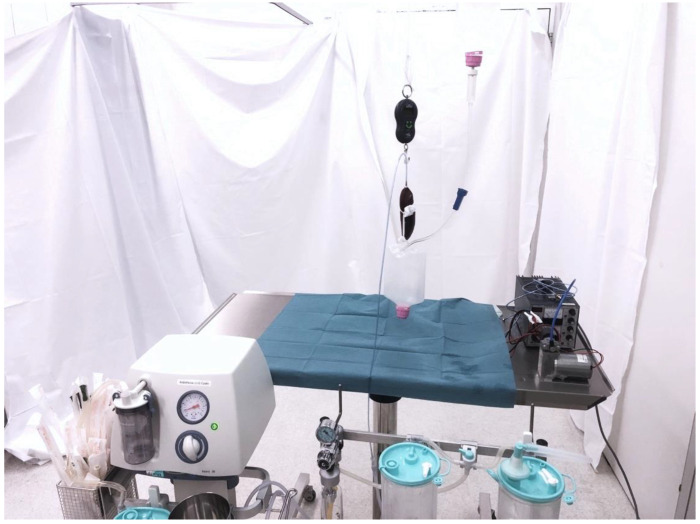
Setup of the holding experiments. The kidney was serially connected with a digital spring balance, completely uplifted, and exposed to additional weight by continuously filling the infusion container attached to the kidney. Negative pressure was provided by a standard OR vacuum suction device.

The first holding force experiments were performed with the “Medela basic 30”, as this device allowed for precise graduated configuration of the vacuum suction force in mmHg (Figure 3). The experiments were repeated five times for each setting. The full results for each setting are shown in Table 1. The best results were achieved with the maximum vacuum suction setting of −600 mmHg, with an average holding force of 316 g across all five tests. It was also noted that a setting of less than −300 mmHg negative pressure was insufficient for the proper functioning of the THD. 

The second holding force experiments were performed with the wall-based “Vakuumregler High-Spatz”. This device allowed only a stepless, numerical-indicator-free adjustment from minimum to maximum; therefore, the measurements were only carried out with the maximum suction force (−600 mmHg). The average holding force was 318 g. This experiment confirmed the adequate function of the THD with vacuum equipment found in every standard OR.

## 4. Discussion

Here, we reported the development and evaluation of a novel THD for laparoscopic and robotic operations, specifically in the context of partial nephrectomy. Our preliminary tests showed that 3D-printed devices made out of stainless steel or polyurethane could interface well with real tissues using conventional laparoscopic or robotic equipment and with standard OR vacuum suction systems. 

Similar suction-based devices have or are being developed for use in other surgical contexts. The Starfish®, OCTOPUS®, and Xpose Access Device® are currently utilized in cardiovascular surgery and allow for safe and effective off-pump coronary artery bypass grafting [9,10,11,12,13] and facilitate the surgical management of mediastinal tumors [9]. The OVALEAD® is a device used in gynecologic surgery to fix the adnexa [14]. A novel lung-stabilizing device for video-assisted thoracoscopic surgery [15,16] and a cornea-holding device for ophthalmologic transplantation surgery [17] using negative pressure are currently under development. To our knowledge, until now, no one has reported on the use of a vacuum-supported device for urological or visceral surgery. 

### 4.1. Limitations 

#### 4.1.1. Material Property

In this study did not assess different material properties, (e.g., soft silicone versus hard metal). However, in the very beginning of the development process and after discussions with our engineers, we actively voted against developing a soft device. The main reason was that a vacuum needs to be maintained on top of a deformable/soft tissue. If both sides (tissue und device) were soft, there would be a risk that the vacuum would not be stable, especially while constantly manipulating and moving the device while on the tumor.

#### 4.1.2. Utility

A key question we anticipated is whether such a device is truly necessary. We would assume that the THD is not mandatory for large or highly exophytic kidney tumors, but we are convinced that the THD could be helpful with endophytic or partially exophytic tumors. With the latter, the tumor could then be pulled virtually out of the tumor bed in a visually delineable higher echelon, leading to better visibility, more traction on the intended resection margins, and with a potentially faster, more precise dissection during warm ischemia time. 

#### 4.1.3. Blood/Clots

Here, we presented a preclinical study using in vitro models of human and porcine kidneys and livers, establishing the foundation for a planned clinical trial. In such a trial, we would need to assess the influence of bleeding and blood clots on the device’s ability to hold and manipulate tissue. It is possible that severe bleeding could impair the THD’s holding abilities. However, severe bleeding usually appears only after significant incision of the kidney parenchyma; this would take place after interfacing the THD with an exophytic tumor. In the event the device is rendered nonfunctional, it can be easily decoupled from the tissue and set aside, allowing the operation to proceed in the conventional manner without impairing the operative field. 

#### 4.1.4. Costs

The costs of the material of the THD are low and in the range of other disposable, non-electronic laparoscopic tools. One of our primary goals for the development process was to keep costs for future applications as low as possible by utilizing equipment already available in every OR, such as a vacuum pump or vacuum connection. This would make such a device’s accessibility, introduction, and distribution straightforward without being particularly cost intensive. 

#### 4.1.5. Ethical Considerations

We sought to preliminarily test our device using real tissues in slaughtered animal specimens as well as cadaveric human tissue. Fixing of the tissue did not appear to significantly alter the device’s ability to suction, hold, and manipulate. Future studies could employ preserved, diseased tissue and live animal models prior to testing in humans. 

## 5. Conclusions

Here, we described a novel and promising vacuum-based tissue holding device in the context of laparoscopic partial nephrectomy. Preliminary in vitro evaluation demonstrated satisfactory device setup, suction, and tissue handling without macroscopic tissue impairment. These results form the basis for future evaluation in an expanded in vivo trial. 

## 6. Patents

Before publication, the THD has been patented (TISSUE HOLDING DEVICE Patent WO2021122618A1) by L.H. and M.G. 

## Figures and Tables

**Table 1:** Results of holding experiments with Medela, Basic 30”, Medela Healthcare, Switzerland (Electric pump).

Setting [mmHg]	Test 1 [g]	Test 2 [g]	Test 3 [g]	Test 4 [g]	Test 5 [g]	Mean [g]
−600	320	350	285	315	310	316
−500	280	270	225	200	215	238
−400	170	140	150	135	140	147
−300	105	110	105	95	110	105
−200	insufficient	insufficient	insufficient	insufficient	insufficient	-

## Data Availability

The data presented in this study are available on request from the corresponding author.

## Supplementary Materials

The following supporting information can be downloaded at: https://www.mdpi.com/article/10.3390/cancers14194618/s1, File S1: Questionnaire VAC-CAP EXPERIMENT.
