# Supplementary material for: Development and In Vitro Assessment of a Novel Vacuum-Based Tissue-Holding Device for Laparoscopic and Robotic Kidney Cancer Operations
Source: Cancers (Basel). 2022 Sep 23;14(19):4618. doi: 10.3390/cancers14194618 (PMC9559531; doi:10.3390/cancers14194618)
Supplement: Supplementary file 1 [file cancers-14-04618-s001.zip › cancers-1885901-supplementary.pdf]

File S1
Questionnaire VAC-CAP EXPERIMENT

Evaluation: Likert scale 1-6 (6 very good)

1. Passes VAC-CAP well through trocar: _____

2. Is the suction/adhesion to the tissue good? __________
- Horizontal movements (sideways, forward/backward)______
- Vertical movements (up/down)______________

3. Are there tissue damages on the surrounding parenchyma? YES / NO

4. Are there are tissue damages on the resected parenchyma (“tumor”)? YES / NO

3. Gripping surface: can the VAC-CAP be gripped well with the instrument?_____

4. Connection to standard tubing: is the connection to the tubing good? ________

5. Does the tubing interfere with the preparation? YES / NO

6. is the tube in the trocar good (only if drained through the trocar?)? YES / NO

7. Can the tube be placed well through the trocar?______

8. Tube: is the tube cause any problems in the trocar? _____

9. Did the resection work well? _______

10. Was the VAC-CAP helpful?________

11. Is the material of the VAC-CAP intact after the procedure? _____

11. Overall impression: ______

Comment:
